# Defense against HSV-1 in a murine model is mediated by iNOS and orchestrated by the activation of TLR2 and TLR9 in trigeminal ganglia

**DOI:** 10.1186/1742-2094-11-20

**Published:** 2014-01-30

**Authors:** Guilherme Pimenta Zolini, Graciela Kunrath Lima, Natália Lucinda, Mariana Almeida Silva, Marcela França Dias, Natália Lima Pessoa, Bruna Pizziolo Coura, Christiane Teixeira Cartelle, Rosa Maria Esteves Arantes, Erna Geessien Kroon, Marco Antônio Campos

**Affiliations:** 1 Laboratório de Imunopatologia, Imunologia de Doenças Virais, Centro de Pesquisas René Rachou, Fundação Oswaldo Cruz, Fiocruz, Av. Augusto de Lima 1715, Belo Horizonte, Minas Gerais 30190-002, Brazil; 2 Escola de Veterinária, Universidade Federal de Minas Gerais, Belo Horizonte, Minas Gerais, Brazil; 3 Departamento de Patologia, Universidade Federal de Minas Gerais, Belo Horizonte, Minas Gerais, Brazil; 4 Departamento de Microbiologia, Universidade Federal de Minas Gerais, Belo Horizonte, Minas Gerais, Brazil

**Keywords:** Encephalitis, HSV-1, Innate immunity, Toll-like receptors, Host immune response

## Abstract

**Background:**

Herpes simplex 1 (HSV-1) causes various human clinical manifestations, ranging from simple cold sores to encephalitis. Innate immune cells recognize pathogens through Toll-like receptors (TLRs), thus initiating the immune response. Previously, we demonstrated that the immune response against HSV-1 is dependent on TLR2 and TLR9 expression and on IFN gamma production in the trigeminal ganglia (TG) of infected mice. In this work, we further investigated the cells, molecules, and mechanisms of HSV-1 infection control, especially those that are TLR-dependent.

**Methods:**

C57BL/6 wild-type (WT), TLR2^−/−^, TLR9^−/−^, and TLR2/9^−/−^ mice were intranasally infected with HSV-1. On the viral peak day, the TG and brains were collected from mice and TLR expression was measured in the TG and brain and inducible nitric oxide synthase (iNOS) expression was measured in the TG by real-time PCR. Immunofluorescence assays were performed in mice TG to detect iNOS production by F4/80^+^ cells. Intraperitoneal macrophages nitric oxide (NO) production was evaluated by the Griess assay. WT, CD8^−/−^, RAG^−/−^, and iNOS^−/−^ mice were intranasally infected in a survival assay, and their cytokine expression was measured in the TG by real-time PCR.

**Results:**

Infected WT mice exhibited significantly increased TLR expression, compared with their respective controls, in the TG but not in the brain. TLR-deficient mice had moderately increased TLR expression in the TG and brain in compare with the non-infected animals. iNOS expression in the WT infected mice TG was higher than in the other groups with increased production by macrophages in the WT infected mice, which did not occur in the TLR2/9^−/−^ mice. Additionally, the intraperitoneal macrophages of the WT mice had a higher production of NO compared with those of the TLR-deficient mice. The CD8^−/−^, RAG^−/−^, and iNOS^−/−^ mice had 100% mortality after the HSV-1 infection compared with 10% of the WT mice. Cytokines were overexpressed in the iNOS^−/−^ infected mice, while the RAG^−/−^ mice were nearly unresponsive to the virus.

**Conclusion:**

TLRs efficiently orchestrate the innate immune cells, eliciting macrophage response (with NO production by the macrophages), thereby controlling the HSV-1 infection through the immune response in the TG of mice.

## Background

Herpesviruses are large enveloped viruses (100–200 nm) with a double-stranded DNA genome from 120 to 230 kbp which encodes approximately 84 proteins [1]. An estimated 70% of the world population is seropositive for human herpes virus 1 (HHV-1), also known as herpes simplex virus 1 (HSV-1) [1,2]. HSV-1 is the etiological agent of orolabial and cutaneous herpes, ophthalmic lesions, keratitis, and kerato-conjunctivitis. Severe ophthalmic lesions, central nervous system involvement (encephalitis), or systemic infections, although rare, can occur in recently born children or in immunosuppressed individuals [3]. Herpesviruses are known for their latency/recurrence phenomena. HSV-1 can become latent in neuronal ganglia (especially the trigeminal ganglia) and may reactivate under stressful conditions, sometimes causing new disease episodes [1].

The HSV-1 infection in humans involves the intimate contact of a susceptible individual with someone who is shedding the virus [1]. After the primary infection, which usually occurs in the oral mucosa, the virus replicates in epithelial cells and then infects the neuronal terminal, from where the virus reaches the nerve ganglia through retrograde axonal flow. The trigeminal ganglia harbor latent virus DNA [4,5]. Recurrences occur when the latent virus reactivates and is transported by anterograde axonal flow to the primary infection site [6,7]. In some cases, reactivated HSV-1 is transported to the central nervous system, causing encephalitis [8]. Reactivation is normally associated with stress factors such as hormonal alterations, ultraviolet light exposure, and immunosuppression [9,10]. However, a study using mathematical models of HSV-2 (which is closely related to HSV-1) reactivation revealed that the virus is constantly released from neurons in small amounts, thereby activating the immune system, which in turn inhibits viral replication and promotes a balance that prevents the clinical reactivation of the disease [11].

Immune responses against HSV-1 involve both innate and acquired immunity. Innate responses are largely mediated by leukocytes such as neutrophils, macrophages, and dendritic cells, which phagocytose pathogens and coordinate additional responses with the synthesis of a variety of inflammatory mediators and cytokines. Toll-like receptors (TLRs) are proteins that confer ‘specificity’ to the innate immune system, allowing the recognition of pathogen associated molecular patterns (PAMPs) [12,13]. The mRNAs from all TLR types (except TLR3) are expressed by monocytes and macrophages [13]; in the central nervous system (CNS), while microglia express mRNA from TLR1 to TLR9, neurons and oligodendrocytes express only TLR3 [12]. During the last decade, the important roles of innate immunity responses and TLRs in HSV-1 infection control have been highlighted [14-19].

HSV-1 infection control appears to be highly dependent on the cellular immune response, including local tissue cells such as microglia and resident macrophages, as well as inflammatory infiltration by cells such as monocytes and CD8^+^ T lymphocytes [19-23]. This control most likely occurs through the production of cytokines such as IFN gamma, especially in the trigeminal ganglia [19,24,25]. IFN gamma is produced mainly by T lymphocytes [26], and one of its major actions is to increase iNOS and ROS production for killing pathogens [26,27]. iNOS, IFN gamma, and other pro-inflammatory cytokines are important for controlling the HSV-1 infection in the trigeminal ganglia through the action of macrophages and CD8^+^ T cells [19,28], although this effect in the brain is controversial [19,28,29].

Data from our group, which uses the murine intranasal infection model, have indicated that the crucial ‘spot’ for immune defense organization against HSV-1 most likely is the trigeminal ganglia and not the brain [14,19,28,30,31]. Most C57BL/6 wild-type (WT) mice infected with 10^6^ p.f.u. of HSV-1 can control the infection and survive [19,31]. These studies have also demonstrated that certain chemokines and IFN gamma expression in trigeminal ganglia exhibit the same temporal profile of viral replication, with peaks of expression occurring at day 5 post infection (which most likely facilitates controlling the virus), while mice without signs of encephalitis do not harbor the virus in their brains [19]. Apparently this control is not as efficient in mice with deficiencies in their immune response because knockout mice for MyD88, IFN gamma [15], TLR9, and TLR2/9 [19] have high mortality rates and die with signs of encephalitis after HSV-1 infection. Additionally, the virus is found in the brains of these animals.

The high prevalence of HSV-1 and the considerably increased number of immunosuppressed individuals due to diseases or to therapeutic transplantation, heighten the importance of comprehending the mechanisms of immune defense against the virus in a herpetic encephalitis model. In the present work, we demonstrate that macrophage-mediated immunity against HSV-1 occurs efficiently through iNOS in trigeminal ganglia and appears to be organized by the initial activation of TLR2 and TLR9 (with a possible interrelation of these receptors with other TLRs), which contributes to viral infection control.

## Materials and methods

### Virus

HSV-1 strain EK [32], which was isolated from a human case of recurrent oral herpes with blisters, was multiplied in Vero cells [15] and purified [33] as previously described. The virus titers obtained were 3.0 × 10^9^ p.f.u./mL.

### Cells

Vero cells (ATCC) were maintained in MEM supplemented with 5% heat-inactivated FBS and antibiotics in 5% CO_2_ at 37°C. These cells were used for multiplication and titration of the virus.

### Mice

TLR2^−/−^ and TLR9^−/−^ mice were generated at Osaka University (Osaka, Japan), and the TLR2/9^−/−^ mice were obtained by crossing the TLR2^−/−^ and TLR9^−/−^ mice at the NIH (USA) and by backcrossing them to the C57BL/6 background for eight generations. These mice were a kind gift of Shizuo Akira and Alan Sher, respectively. C57BL/6 RAG^−/−^, C57BL/6 iNOS^−/−^, and C57BL/6 CD8^−/−^ mice were acquired from Biotério Fiocruz/Rio. The C57BL/6 (WT, control) and knockout mice were maintained in a pathogen-free, barrier environment in Centro de Pesquisas René Rachou, Oswaldo Cruz Foundation (CPqRR/FIOCRUZ) (Belo Horizonte, Minas Gerais, Brazil). Six- to 10-week-old male mice were anesthetized with ketamine (Agribrands do Brasil Ltda, Brazil), and 10^6^ p.f.u. of the purified HSV-1 contained in 10 μL was inhaled by the mice as described previously [34]. The control mice inhaled PBS. The mouse colonies and all of the experimental procedures were performed according to the institutional animal care and use guidelines from CPqRR/FIOCRUZ. The project was previously approved by the Ethics Committee in Animal Experimentation (CEUA from CPqRR/FIOCRUZ LW6/11).

•Intraperitoneal macrophages

Thioglycollate-elicited peritoneal macrophages were obtained from either C57BL/6, TLR2^−/−^, TLR9^−/−^, or TLR2/9^−/−^ mice by peritoneal washing, were activated with sub-optimal concentration of murine IFN gamma (50 U/mL) as previously described [15] and then were stimulated with HSV-1 (MOI of 10) for 24 h. The Griess reaction assay [35] was performed to evaluate nitric oxide production in the supernatants.

### Griess reaction assay

Thioglycollate-elicited peritoneal macrophages were obtained from either C57BL/6, TLR2^−/−^, TLR9^−/−^, or TLR2/9^−/−^ mice by peritoneal washing. Adherent peritoneal macrophages were cultured in 96-well plates (2 × 10^5^ cells/well) at 37°C/5% CO_2_ in Dulbecco’s modified Eagle’s medium (Life Technologies, Paisley, UK) supplemented with 5% heat-inactivated fetal bovine serum (Life Technologies), 2 mmol/L L-glutamine (Sigma) and 40 μg/mL of gentamicin (Schering do Brasil, Rio de Janeiro, Brazil). Next, the cells were stimulated with HSV-1 (multiplicity of infection, 10), for 24 h to evaluate nitric oxide production in the supernatants.

### RNA extraction

Trigeminal ganglia and brains were aseptically removed and stored at −70°C until processing. RNA extraction was performed according to the procedures provided by the manufacturer of TRIzol reagent (Invitrogen, USA). The RNAs were treated with DNase (Biolabs, USA). One microliter of the extracted RNA was quantified in a Nanodrop ND-1000 spectrophotometer, at wavelengths of 260 and 280 nm.

### Reverse transcription

Reverse transcription was performed according to the procedures provided by the manufacturer of the M-MLV RT enzyme (Promega, USA).

### Real-time PCR

Real-time quantitative PCR (Applied Biosystems, USA) was performed to verify the mRNA expression in the trigeminal ganglia and brain of mice infected with HSV-1. The reactions were performed using the Sybr Green PCR Master Mix (Applied Biosystems, USA) in the Applied Biosystems’ 7000 Sequence Detection System, at 50°C, 2’; 95°C, 10’; and 40 cycles of 95°C, 15” and 60°C, 1’, followed by a final dissociation stage. The oligonucleotides used in the reactions were specific for HPRT, **F -** GTT GGA TAC AGG CCA GAC TTT GTT G and **R -** GAT TCA ACT TGC GCT CAT CTT AGG C; IP-10 (CXCL10), **F -**GCC GTC ATT TTC TGC CTC AT and **R -** GCT TCC CTA TGG CCC TCA TT; TNF alpha, **F -** CAT CTT CTC AAA ATT CGA GTG ACA A and **R -** TGG GAG TAG ACA AGG TAC AAC CC; iNOS, **F -** CAG CTG GGC TGT ACA AAC CTT and **R -** CAT TGG AAG TGA AGC GTT TCG; MCP-1(CCL2), **F -** CTT CTG GGC CTG CTG TTC A and **R -** CCA GCC TAC TCA TTG GGA TCA [36]; RANTES (CCL5), **F -** GTC GTG TTT GTC ACT CGA AGG A and **R -** GAT GTA TTC TTG AAC CCA CTT CTT CTC; gp91, **F -** CCA ACT GGG ATA ACG AGT TCA AGA C and **R -** AAG GCT TCA GGG CCA CAC A; p22, **F -** TGG CTA CTG CTG GAC GTT TCA C and **R -** CTC CAG CAG ACA GAT GAG CAC AC; VP16 **F -** TTT GAC CCG CGA GAT CCT AT and **R -** GCT CCG TTG ACG AAC ATG AA [37]; TLR1, **F -** TGA TCT TGT GCC ACC CAA CA and **R -** GCA GGG CAT CAA AGG CAT TA; TLR2, **F** - TTG CTC CTG CGA ACT CCT AT and **R** - AGC CTG GTG ACA TTC CAA GA; TLR3, **F -** TAG ACT GCA TCG CCT GCT AA and **R -** AAG CAG CCA GAA GCA GAA CT; TLR6, **F -** TCT GGG ATA GCC TCT GCA ACA and **R -** GGC GCA AAC AAA GTG GAA AC; TLR7, **F -** TAC CAG GAC AGC CAG TTC TA and **R -** AGG AGC CTC TGA TGA GAC AA; and TLR9, **F** - ACC TCA GCC ACA ACA TTC TC and **R** - TGC ACC TCC AAC AGT AAG TC [38]. The comparative Ct method was chosen to analyze the data, using the arithmetic formula 2^-ΔΔCt^[39]. The genes expression was normalized to expression of the constitutively expressed gene Hypoxanthine-guanine phosphoribosyltransferase (HPRT). All of the reactions were replicated.

### Histopathology

Samples were fixed with 10% formaldehyde in phosphate buffer, routinely processed and embedded in paraffin, as previously described [15].

### Histopathology and immunostaining

For trigeminal ganglia immunostaining, samples were frozen in the Tissue-Tek O.C.T. compound (Sakura, Netherlands), and 5 μm slices were cut using a HM505N microtome cryostat (Microm, USA). The tissues were stained as previously described [40], with modifications. Immediately thereafter, primary antibodies were incubated for 2 h, washed, and incubated with a labeled secondary antibody. The sections were counterstained (nuclei) by Hoechst (0.2 μg/mL) (Molecular Probes, USA) and mounted in the Hydromount aqueous medium (National Diagnostics, USA). The primary antibody used was rat anti-F4/80, 1:50 (Serotec MCAP497, USA) or rabbit anti-iNOS, 1:5,000 (Spring Bioscience E3740, USA).

The secondary antibody was Alexa Fluor 488 goat anti-rat IgG (1:500) (Molecular Probes, USA) or goat anti-rabbit IgG (1:500) (Molecular Probes, USA). The Immunostains were observed and photographed via an Olympus BX51 microscope (Olympus, Japan) using a Megacybernetics color digital camera and Image Pro-Express software.

### Morphometry

We quantified at least three fields per sample (*n* = 3/group). Each field generated three superimposed images obtained under a microscope Olympus BX51 (Olympus, Tokyo, Japan), using three fluorescence filters (iNOS, F4/80, Hoechst). The images were transferred by video camera Cool SNAP-colored Procf Color (Media Cybernetics, Bethesda, MD, USA) to a computer system coupled to video using the Image program-Pro Express version 4.0 for windows (Media Cybernetics, Bethesda, MD, USA). Quantification of total cells (Hoechst) and iNOS positive cells and F4/80 markers was performed with the aid of the ImageJ software (version 1:46).

### Statistical analysis

The analyses were performed using the GraphPad Prism 5 software for Windows (GraphPad Software, Inc., La Jolla, CA, USA). The sample groups were assessed by non-parametric or parametric tests, according to the Kolmogorov-Smirnov normality. Real-time PCR results were statistically analyzed using Mann–Whitney non-parametric T-tests. Macrophage results were analyzed by unpaired T-tests. For the survival curves, the statistical analyses were performed using the log-rank values.

## Results and discussion

### Expression of TLRs in HSV-1 infected mice TG and brains occurs in a TLR-interdependent manner

Inter-relationships between the TLR gene expression levels were already evidenced by previous research in which mice pre-stimulated with a TLR3 agonist, poli I:C, exhibit increased TLR2 expression in the brains and reduced mortality after the HSV-1 infection [41]. Although the importance of TLR2 and TLR9 in the HSV-1 infection is well described in the literature [14-19], their inter-relationship with other TLRs has been poorly explored. To investigate this inter-relationship, we analyzed the expression of several TLRs in trigeminal ganglia (TG) and brains of WT and knockout mice for TLR2, TLR9, and TLR2/9, after infection with HSV-1, to determine whether the deaths of TLR2/9^−/−^ mice [19] after HSV-1 infection occur exclusively because of the absence of these specific TLRs or whether their absence could interfere with the expression and/or recognition through other TLRs.

WT, TLR2^−/−^, TLR9^−/−^, and TLR2/9^−/−^ mice were intranasally infected with 10^6^ p.f.u. of HSV-1 [19] and were euthanized on the fifth day post infection (day of the TG viral peak) [19], and the TG and brains were collected to evaluate the TLR expression by real-time PCR.

**Figure 1:**
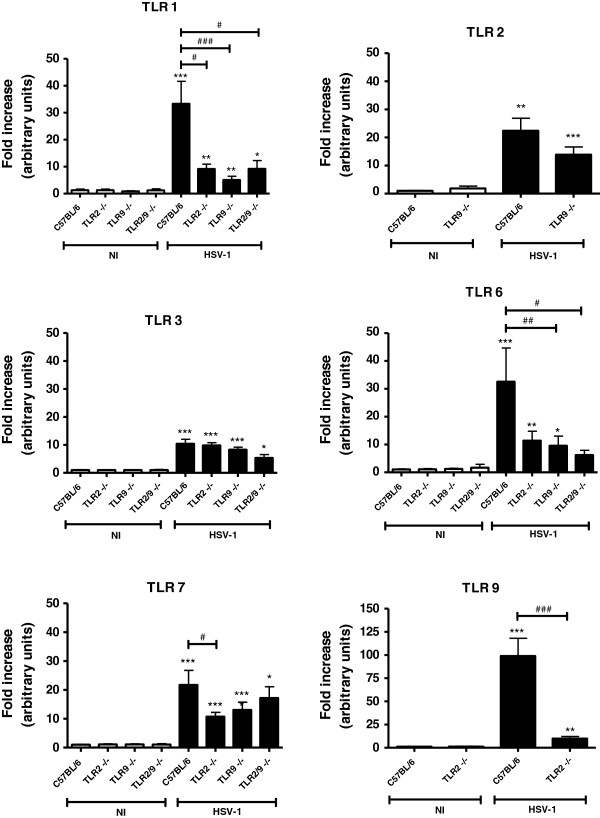
**TLR expression in the mouse trigeminal ganglia.** Mice were either infected intranasally with 10^6^ p.f.u. of HSV-1 or were not infected, and they were euthanized 5 days thereafter. The trigeminal ganglia were collected, and the RNA was extracted, reverse transcribed in cDNA and real-time PCR for TLR expression indices for C57BL/6 or the indicated knockout (NI) (*n* = 4) or the respective infected groups (*n* = 6 to 9) were obtained (*P <0.05; **P <0.01; ***P <0.001 when comparing the infected animals with the respective non-infected animals); (#*P* <0.05, ##*P* <0.01, ###*P* <0.001, when comparing the infected C57BL/6 mice with the infected knockout mice). The results are representative of two experiments that yielded similar results.

**Figure 2:**
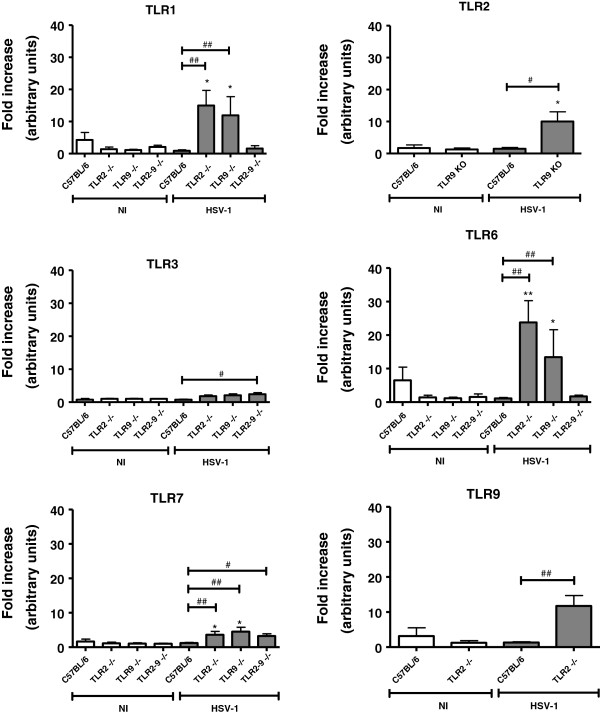
**TLRs expression in mice brains.** Mice were either infected intranasally with 10^6^ p.f.u. of HSV-1 or not infected and they were euthanized 5 days thereafter. The brains were collected, and the RNA was extracted, reverse transcribed in cDNA and real-time PCR for TLR expression indices for C57BL/6 or the indicated knockout (NI) (*n* = 4) or the respective infected groups (*n* = 6 to 9) were obtained (*P <0.05; **P <0.01, when comparing the infected animals with the respective non-infected animals); (#*P* <0.05; ##*P* <0.01, when comparing the infected C57BL/6 mice with the infected knockout mice). The results shown are representative of two experiments that yielded similar results.

Data of the TLR expression in the WT infected mice revealed that TLRs are highly expressed in the TG (Figure 1), but there was no increase in TLR expression in the brain (Figure 2), thereby demonstrating that the innate immunity against HSV-1 in this model occurs preferentially in the TG. *In vitro* assays from another research group [42] have revealed the synergic action of IFN gamma and IFN alpha/beta in controlling HSV-1 infection. These cytokines genes are activated by phosphorylation cascades that, in turn, are initiated by different TLRs, and this synergistic action impairs viral replication.

The remaining results of this assay (Figures 1 and 2) indicate that TLRs 1, 2, 3, 6, 7, and 9 are expressed in the TG of WT mice infected with HSV-1. Expression of TLR1, TLR2 (except for the TLR2^−/−^ and TLR2/9^−/−^ mice), TLR3, TLR6, TLR7, and TLR9 (except for the TLR9^−/−^ and TLR2/9^−/−^ mice) in the TG (Figure 1), was generally diminished in TLR knockout mice compared with WT mice. These results suggest an interaction of different TLRs for immune-response orchestration in the TG of WT mice. In the knockout mouse brains (Figure 2), there was an increase or a tendency toward an increase in TLR1 (except for the TLR2/9^−/−^ mice), TLR2 (except for the TLR2^−/−^ and TLR2/9^−/−^ mice), TLR3, TLR6 (except for the TLR2/9^−/−^ mice), TLR7, and TLR9 (except for the TLR9^−/−^ and TLR2/9^−/−^ mice) expression. These results suggest an interaction of different TLRs for immune-response orchestration in the brains after a deficient response in the TG.

When HSV-1 recognition by TLRs in the TG failed (in the case of certain TLR-knockout mice), there was a weak immune response in the brains. In TLR2^−/−^ infected mice (which have a mortality rate of 10% [19], similar to WT) this brain response appears efficiently control the virus, thereby reinforcing the fundamental role of TLR9 (which is expressed in these animal brains). By contrast, mice that do not express TLR9 (that is, TLR9^−/−^ and TLR2/9^−/−^ mice) cannot control the virus and die, although they express other TLRs in their brains. Double knockout mice (TLR2/9^−/−^) not only had diminished expression of TLR in their TG but also did not express TLR1 and TLR6 in their brains, most likely caused by difficulty of the immune system in orchestrating a compensatory defense without the functional presence of TLR2 and TLR9, which is in accordance with the mortality rate of 100% in the TLR2/9^−/−^ group [19].

Another relevant piece of data is the significant rise in the IFN gamma and IL-1 beta expression in the brain of TLR2^−/−^ compared with their non-infected controls [19]. These cytokines are crucial for an efficient immune response in the TG of WT infected mice [19]. Although the TLR2^−/−^ mice had a mortality rate similar to WT mice when infected by HSV-1, the former had lower levels of TLR1 and TLR7 expression in the TG but higher levels of TLR1, TLR3, TLR6, TLR7, and TLR9 expression in the brain. Taken together, these results corroborate the hypothesis that TLR2^−/−^ mice are capable of mounting a compensatory immune response in the brain, despite the deficient response in the TG.

### The expression of iNOS and NO production are TLR-dependent during HSV-1 infection

Our previous experiments indicated the importance of macrophages, CD8^+^ T lymphocytes and IFN gamma [19] for HSV-1 infection control in the murine model of intranasal infection. IFN gamma production by CD8^+^ T lymphocytes could be fundamental to macrophage activation in trigeminal ganglia in these conditions, increasing the reactive oxygen species (ROS) and reactive nitrogen species (RNS) production by this type of cell [26]. Subsequently, we investigated which mechanisms of monocytes/macrophages responses are involved in HSV-1 control.

To this end, the TG of WT and knockout mice were collected 5 days after intranasal infection, and the iNOS, gp91, and p22 expression was evaluated by real-time PCR. iNOS is the enzyme responsible for NO production and, consequently, RNS [27]. gp91 ^phox^ and p22 ^phox^ are NADPH oxidase subunits that are membrane-bound protein components of flavocytochrome b558, which is responsible for electron transport [43,44] and ROS production.

**Figure 3:**
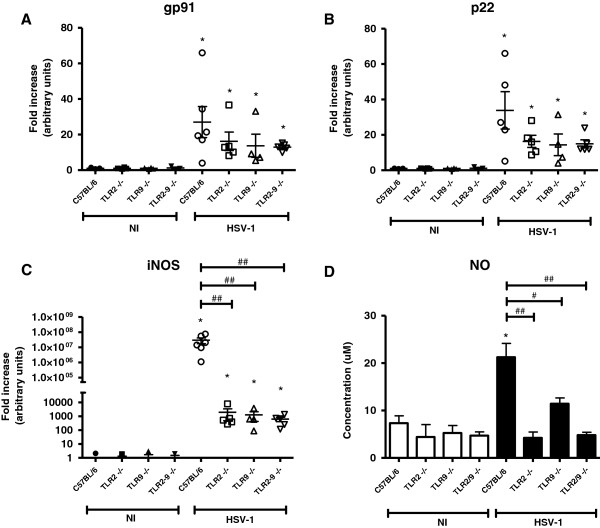
**iNOS, gp91, and p22 expression in the trigeminal ganglia of the infected mice and NO production in the infected peritoneal macrophages.** Mice were infected intranasally with 10^6^ p.f.u. of HSV-1 and were euthanized 5 days after the infection. The trigeminal ganglia were collected, the RNA extracted, reverse transcribed in cDNA and real-time PCR for the expression of **(A)** gp91^phox^, **(B)** p22^phox^, and **(C)** iNOS gene were performed. **(D)** Macrophages derived from the C57BL/6 mice or the indicated knockout mice were infected with HSV-1 (MOI of 10, five wells/group) (black bars) or were non-infected (NI, white bars), and the levels of nitric oxide (NO) were determined in the supernatants, 24 h after stimulation, by the Griess reaction assay. The bars represent the S.E.M. (*P <0.05, when comparing the infected macrophages to the respective non-infected macrophages; #*P* <0.05 or ##*P* <0.01 when comparing the C57BL/6 infected macrophages with the knockout infected macrophages. This experiment is representative of two analyses.

The infected-mouse groups displayed a significant increase in gp91^phox^ and p22^phox^ compared with the respective non-infected controls (Figure 3A and B, respectively). Furthermore although the WT infected mice expressed these genes with a tendency toward higher levels than in the knockout mice, the difference between them was not statistically significant. iNOS expression was increased in WT and knockout infected compared with non-infected mice, with a more pronounced expression in the WT mice than in the other groups (Figure 3C). iNOS effectively contributes to the HSV-1 defense, and our results indicate that TLRs are important for iNOS activation/production, while ROS apparently do not play a relevant role in this model’s TLR-dependent response.

To confirm the TLR importance for NO production, peritoneal macrophages of WT, TLR2^−/−^, TLR9^−/−^, and TLR2/9^−/−^ mice were stimulated with HSV-1 (MOI = 10), and the NO production was evaluated by the Griess reaction (Figure 3D). The WT infected-mouse group presented higher amounts of NO compared with the controls and the knockout-infected mouse groups. This result corroborates publications that indicate the importance of iNOS in the defense against HSV-1 and the ability of NO to inhibit HSV-1 replication *in vitro*[21,45,46]. Moreover, this finding confirms the importance of TLR2 and TLR9 in NO production.

**Figure 4:**
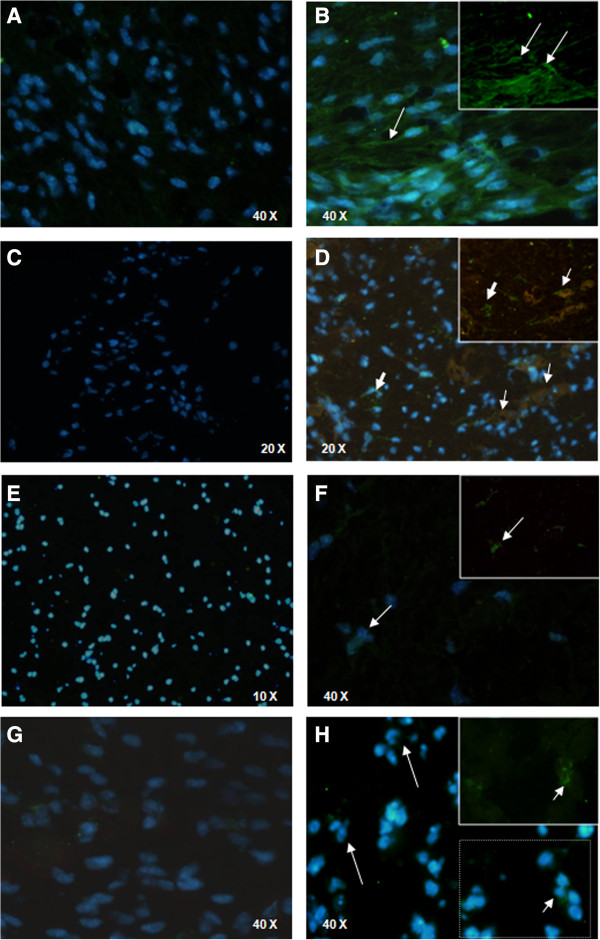
**NO production by F4/80**^**+ **^**cells in the trigeminal ganglia of the infected mice.** C57BL/6 **(A, B, C, D)** or TLR2/9^−/−^**(E, F, G, H)** mice were either inoculated with 10^6^ p.f.u. of HSV-1 or with PBS (uninfected) via the intranasal route; on the fifth d.p.i., the trigeminal ganglia were collected and processed for analysis. **(A)** anti-iNOS Ab (green), C57BL/6 uninfected (400×); **(B)** anti-iNOS Ab (green), C57BL/6 infected (400×); **(C)** anti-iNOS Ab (red) plus anti-F4/80 Ab (green), C57BL/6 uninfected (100×); **(D)** anti-iNOS Ab (red) plus anti-F4/80 Ab (green), C57BL/6 infected, (200×); **(E)** anti-iNOS Ab (green), TLR2/9^−/−^ uninfected, (100×); **(F)** anti-iNOS Ab (green), TLR2/9^−/−^ infected (400×); **(G)** anti-iNOS Ab (red) plus anti-F4/80 Ab (green), TLR2/9^−/−^ uninfected (400×); (H) anti-iNOS Ab (red) plus anti-F4/80 Ab (green), TLR2/9^−/−^ infected (400×). All of the nuclei were stained with Hoechst (blue). Thin arrows **(B**, insert in **B**, **F**, insert in **F)** indicate stained iNOS cells (green); block arrows **(D)** indicate staining only for F4/80 (green) and thin arrows **(D)** indicate double staining for F4/80 and iNOS (brown). Short arrows **(H)** indicate staining for F4/80 (green), and long thin arrows **(H)** indicate stained nuclei (blue) characteristic of polymorphonuclear leukocytes.

Additionally, immunofluorescence assays were performed to visualize iNOS production by F4/80^+^ cells in the TG. In the single-staining assays, TG slices were incubated with antibodies against iNOS stained with Alexa Fluor 488 (green) and were counterstained with Hoechst (blue) for nuclei visualization (Figures 4A, B, E, F). In the double-staining assays, TG slices were incubated with antibodies against F4/80 stained with Alexa Fluor 488 (green) and antibodies against iNOS stained with Alexa Fluor 546 (red), and then counterstained with Hoechst (Figures 4C, D, G, H). The panels in (Figure 4) display the details of the ganglia cell immunofluoresce.

There was no iNOS production in the single-stained cells of the non-infected mouse TG (Figure 4A and E - green) or in the double-stained cells (Figures 4C and G - red). The WT infected mice (Figure 4B) had thin cytoplasmic prolongations stained in green (insert). Most of the WT mouse cells (blue nuclei) expressed the enzyme (Figure 4B), and there was a considerable superposition of F4/80 and iNOS in the double-stained cells (green + red = brown) (Figure 4D), indicating that monocytic cells are the main producers of iNOS in the TG. Some F4/80 positive cells did not express iNOS (thick arrows, Figure 4D); however, many cells were double stained (insert, arrow).

In the TLR2/9^−/−^ infected mice, the iNOS cell staining was modest (Figure 4F - long arrows). Although the inflammatory infiltration was conspicuous (Additional file 1: Figure S1 and Additional file 2: Commentary on the results and discussion in Additional file 1: Figure S1), the cells were predominantly polymorphonucleated, with segmented nuclei, and a few macrophages were stained with F4/80 (Figure 4H - long arrows). Few mononuclear F4/80 positive cells were visible in the TG slices from the TLR2/9^−/−^ mice (Figure 4H - insert, arrows), and iNOS-stained cells were rare (Figure 4H - selection).

Altogether, the TG immunofluorescence revealed a greater iNOS production by F4/80 positive cells in the WT infected mice (Figure 4B and D), in comparison to TLR2/9^−/−^ (Figure 4F and H), which have a high mortality rate when infected by HSV-1 [19]. The morphometry (Additional file 3: Figure S2 and Additional file 2: Commentary on the results and discussion in Additional file 3: Figure S2) did not show statistically significant difference in the production of iNOS, in the amount of macrophages nor in the production of iNOS by macrophages between infected C57BL/6 and infected TLR2/9^−/−^ mice. However, there was a clear trend towards higher levels of iNOS, more macrophages, and more iNOS producer macrophages in infected C57BL/6 mice than in infected TLR2/9^−/−^ mice. We therefore believe that this trend is biologically relevant.

### Macrophages and lymphocytes are essential for controlling HSV-1 infection

**Figure 5:**
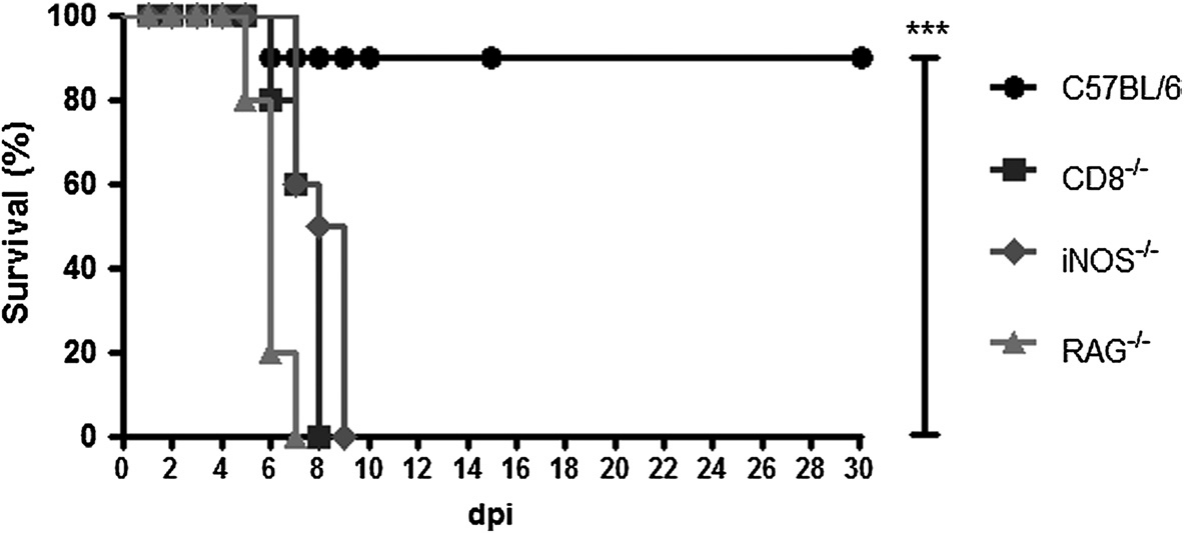
**Survival rates of the CD8**^**−/−**^**, RAG**^**−/−**^, **and iNOS**^**−/− **^**mice after the HSV-1 infection.** Mice were inoculated with 10^6^ p.f.u. of HSV-1 (*n* = 10 for each group), and the mouse mortality was measured daily. C57BL/6 (circle), CD8^−/−^ (square), iNOS^−/−^ (diamonds), and RAG^−/−^ (triangle). The statistical analyses were performed using the log-rank test. ***P <0.001, when comparing survival from C57BL/6 to each group of knockout mice. This experiment is representative of two analyses.

To confirm the importance of lymphocytes and macrophages against the HSV-1 infection in the experimental model of herpetic encephalitis, RAG^−/−^, iNOS^−/−^, CD8^−/−^, and WT mice (*n* = 10 from each group) were intranasally infected with 10^6^ p.f.u. of HSV-1 [19] to evaluate the mortality (Figure 5), and the control mice aspirated only PBS. The animals were checked daily for encephalitis signs such as prostration, ruffled fur, limb paralysis, and hunched posture, and mice showing these signs were euthanized.

All of the control mice survived, and the WT mice exhibited 90% survival (Figure 5); no CD8^−/−^, RAG^−/−^, or iNOS^−/−^ mice survived (Figure 5). These results indicate that not only T and B lymphocytes [15,19] but also iNOS production is essential to control the viral infection, in accordance with the results reported by other researchers [21] who have demonstrated the importance of NO for preventing HSV-1 replication in the TG.

**Figure 6:**
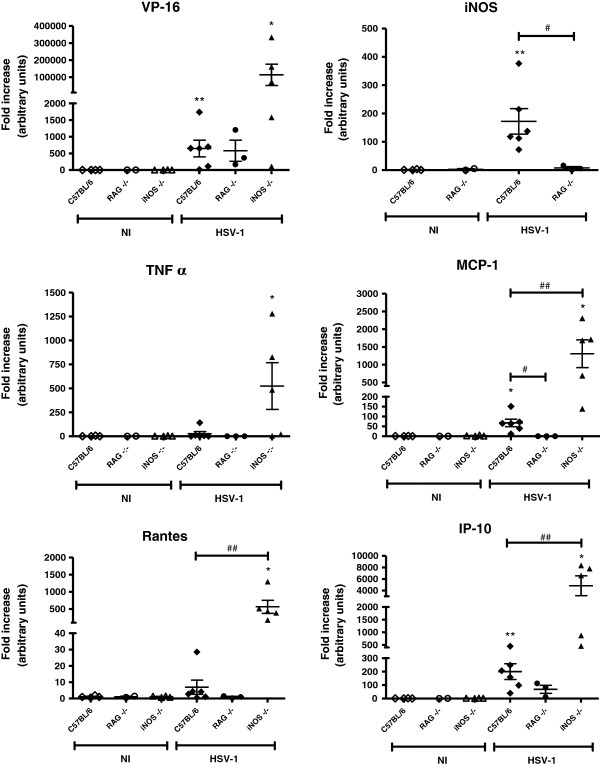
**Virus transcript, inos and cytokine expression in the trigeminal ganglia of the RAG**^**−/− **^**and iNOS**^**−/− **^**mice*.*** The C57BL/6, RAG^−/−^, and iNOS^−/−^ mice were inoculated either with 10^6^ p.f.u. of HSV-1 or with PBS (uninfected) by the intranasal route; on the fifth d.p.i., the trigeminal ganglia were collected and processed for analysis to verify the expression of the viral gene VP-16 and the *inos*, *tnf alpha*, *mcp-1*, *rantes*, and *ip-10* genes. *P <0.05; *P* <0.01, when comparing the infected mice with the respective non-infected mice. #*P* <0.05; ##*P* <0.01, when comparing the C57BL/6 infected mice with the indicated infected knockout mice. The displayed results are representative of two experiments that yielded similar findings.

Additionally, the WT, RAG^−/−^ and iNOS^−/−^ mice’s cytokine and viral transcript expression was evaluated in the TG by real-time PCR, 5 days after the infection. The viral transcripts were similarly expressed in all of the infected mice, but the RAG^−/−^ mice were nearly irresponsive to the virus in terms of their chemokines, effector cytokine (TNF alpha) and iNOS expression (Figure 6). The chemokines MCP-1, Rantes, and IP-10, which attracts immune cells to the infection site, as well as the effector cytokine TNF alpha, were over-expressed in the iNOS^−/−^ infected mice compared with the WT mice. Because all of the infected iNOS^−/−^ mice died (Figure 5), we can assume that, although murine cells can use chemokines to recruit immune cells to the infection site, these immune cells fail to combat the virus because they are not able to produce nitric oxide. In the most likely compensatory mechanism, the cells produce more chemokines to attract additional immune cells, which produce more TNF alpha; however, this process is not sufficient to control the HSV-1 infection.

In summary, we conclude that, subsequent to infection by HSV-1, TLRs efficiently organize the innate immune cells, thereby eliciting a CD8^+^ T cell [19] and macrophage response (with NO production by the macrophages) to control the HSV-1 infection in the mouse TG, thereby preventing severe disease outcomes, such as encephalitis and death.

## Competing interests

The authors declare that they have no competing interests.

## Authors’ contributions

GPZ, GKL, RMEA, EGK, and MAC conceived and designed the experiments. GPZ, GKL, RMEA, NL, MGAS, MFD, NLP, BPC, and CTC performed the experiments. GPZ and GKL performed the statistical analyses. GPZ, GKL, RMEA, EGK, and MAC analyzed the data. RMEA, EGK and MAC contributed reagents, materials, and analysis tools. GPZ, GKL, RMEA, EGK, and MAC wrote the paper. All the authors have read and approved the final version of the manuscript.

## Supplementary Material

Additional file 1: Figure S1 The animals were intranasally inoculated with 10^6^ p.f.u. of HSV-1 or PBS (control). On the fifth d.p.i., the trigeminal ganglia were collected, and representative sections were processed for histological analysis (H&E stained sections). Uninfected (A) and infected C57BL/6 mice (B); uninfected (C) and infected TLR2^−/−^ mice (D); uninfected (E) and infected TLR9^−/−^ mice (F); uninfected (G) and infected TLR2/9^−/−^ mice (H). The architecture and cellularity appeared normal in the uninfected groups. All of the infected animals displayed inflammatory characteristics, with increased cellularity and histologically evident vascular phenomena (edema) and inflammatory changes. The higher intensity of diffuse infiltration by polymorphonuclear cells was accompanied by degenerative neuronal phenomena in the TLR9^−/−^ (F) and TLR2/9^−/−^ (H) mouse groups, compared with the other groups of mice. The lower intensity of inflammation occurred in the C57BL/6 mouse group (B). Original magnifications, 100×; inserts 400 × . Click here for file

Additional file 2 **Supplementary Material.** Supplementary statistics data from Figures 1 and 2. Commentary on the results and discussion in Additional files 1 and 3: Figure S1 and S2. Click here for file

Additional file 3: Figure S2 Morphometry. For quantification of the immunostained cells, C57BL/6 (*n* = 5) or TLR2/9^−/−^ (*n* = 5) mice were infected and after 5 days they were euthanized, the trigeminal ganglia were collected and processed for analysis and graphic comparison of the fluorescence, as stated in materials and methods. (A) anti-iNOS Ab; (B) anti-F4/80 Ab; (C) anti-iNOS plus anti-F4/80 Abs. Click here for file
