# Supplementary figures and images for: Defense against HSV-1 in a murine model is mediated by iNOS and orchestrated by the activation of TLR2 and TLR9 in trigeminal ganglia
Source: J Neuroinflammation. 2014 Jan 30;11:20. doi: 10.1186/1742-2094-11-20 (PMC3922087; doi:10.1186/1742-2094-11-20)

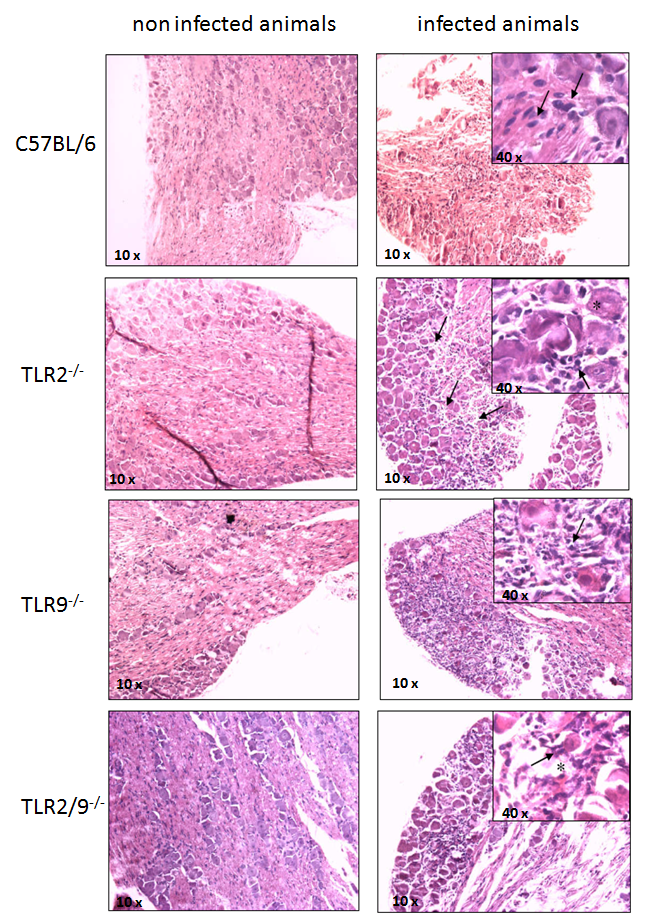

Supplement: Additional file 1: Figure S1 — The animals were intranasally inoculated with 106 p.f.u. of HSV-1 or PBS (control). On the fifth d.p.i., the trigeminal ganglia were collected, and representative sections were processed for histological analysis (H&E stained sections). Uninfected (A) and infected C57BL/6 mice (B); uninfected (C) and infected TLR2−/− mice (D); uninfected (E) and infected TLR9−/− mice (F); uninfected (G) and infected TLR2/9−/− mice (H). The architecture and cellularity appeared normal in the uninfected groups. All of the infected animals displayed inflammatory characteristics, with increased cellularity and histologically evident vascular phenomena (edema) and inflammatory changes. The higher intensity of diffuse infiltration by polymorphonuclear cells was accompanied by degenerative neuronal phenomena in the TLR9−/− (F) and TLR2/9−/− (H) mouse groups, compared with the other groups of mice. The lower intensity of inflammation occurred in the C57BL/6 mouse group (B). Original magnifications, 100×; inserts 400 × . [file 1742-2094-11-20-S1.tiff]

## Slide 1
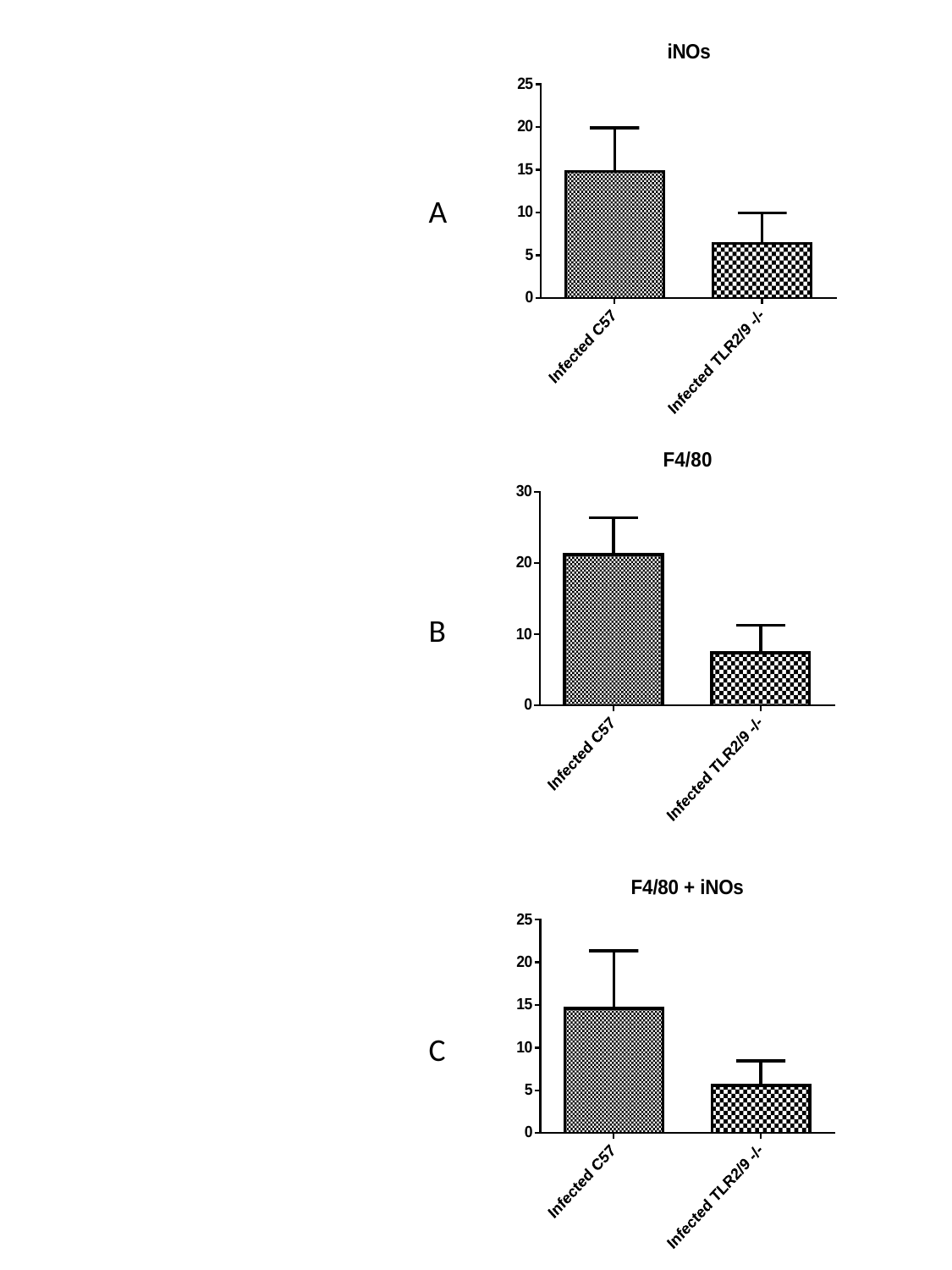

A
B
C

Supplement: Additional file 3: Figure S2 — Morphometry. For quantification of the immunostained cells, C57BL/6 (n = 5) or TLR2/9−/− (n = 5) mice were infected and after 5 days they were euthanized, the trigeminal ganglia were collected and processed for analysis and graphic comparison of the fluorescence, as stated in materials and methods. (A) anti-iNOS Ab; (B) anti-F4/80 Ab; (C) anti-iNOS plus anti-F4/80 Abs. [file 1742-2094-11-20-S3.pptx]
