# Supplementary material for: Defense against HSV-1 in a murine model is mediated by iNOS and orchestrated by the activation of TLR2 and TLR9 in trigeminal ganglia
Source: J Neuroinflammation. 2014 Jan 30;11:20. doi: 10.1186/1742-2094-11-20 (PMC3922087; doi:10.1186/1742-2094-11-20)
Supplement: Additional file 2 — Supplementary Material. Supplementary statistics data from Figures 1 and 2. Commentary on the results and discussion in Additional files 1 and 3: Figure S1 and S2. [file 1742-2094-11-20-S2.doc]

**SUPPLEMENTARY MATERIAL**

Supplementary statistics data from Figures 1 and 2

In the assay to determine the expression of TLR1 in the trigeminal ganglia (Figure 1) of the C57BL/6 WT (WT), TLR2^-/-^, TLR9^-/-^ and TLR2/9^-/-^ infected mice, the arbitrary units were 33.3±8.3, 9.2±1.8, 5.1±1.4 and 9.2±3.1, respectively. The values were significantly different when comparing the WT and knockout groups with their respective non-infected groups; and were significantly different between the WT and knockout infected groups. For the brain TLR1 expression (Figure 2) in the same groups, the values were 0.9±0.3, 15,0±4.7, 11.9±5.8 and 1.6±0.9, respectively. Significant differences were found between the TLR2^-/-^ and TLR9^-/-^ mouse groups and their respective negative control groups, as well as between the WT, TLR2^-/-^ and TLR9^-/-^ infected mouse groups.

In the assay to determine the expression of TLR2 in the trigeminal ganglia (Figure 1) of the WT and TLR9^-/-^ infected mice, the arbitrary values were 22.4±4.5 and 13.9±2.8, respectively. A significant difference was demonstrated between the WT and TLR9^-/-^ mouse groups and their respective negative control groups. The brain TLR2 values (Figure 2) in the same mouse groups were 1.5±0.4 and 10.0±3.0, respectively. Significant differences were found between the TLR9^-/-^ mouse groups and their respective negative control groups, as well as between the WT and TLR9^-/-^ infected mouse groups.

In the assay to determine the TLR3 expression in the trigeminal ganglia (Figure 1) of the WT, TLR2^-/-^, TLR9^-/-^ and TLR2/9^-/-^ infected mice, the arbitrary units were 10.4±1.6, 9.8±1.0, 8.3±0.9 and 5.3±1.2, respectively. Significant differences were observed between the WT and knockout groups and their respective negative control groups. In the brains (Figure 2) of the same groups, the values were 0.8±0.2, 1.8±0.4, 2.1±0,4 and 2.4±0.5, respectively. Significant differences were observed between the WT and TLR2/9^-/-^ infected mouse groups.

In the assay to determine the TLR6 expression in the trigeminal ganglia (Figure 1) of the WT, TLR2^-/-^, TLR9^-/-^ and TLR2/9^-/-^ infected mice, the arbitrary units were 32.6±12.1, 11.4±3.4, 9.5±3.5 and 6.2±1.7, respectively. Significant differences were observed between the WT, TLR2^-/-^ and TLR9^-/-^ mouse groups and their respective negative control groups, as well as between the WT, TLR9^-/-^ and TLR2/9^-/-^ infected mouse groups. In the brains (Figure 2) of the same mouse groups, the values were 1.1±0.2, 23.8±6.5, 13.4±8.2 and 1.7±0.4, respectively. Significant differences were observed between the TLR2^-/-^ and TLR9^-/-^ mouse groups and their respective negative control groups, as well as between the WT, TLR2^-/-^ and TLR9^-/-^ infected mouse groups.

The arbitrary units for TLR7 expression in the trigeminal ganglia (Figure 1) of the WT, TLR2^-/-^, TLR9^-/-^ and TLR2/9^-/-^ infected mice were 21.7±5.0, 10.7±1.5, 13.1±2.7 and 17.2±3.9, respectively. Significant differences were observed between the WT, TLR2^-/-^ and TLR9^-/-^ mouse groups and their respective negative control groups, as well as between the WT and TLR2^-/-^ infected mouse groups. In the brains (Figure 2) from the same groups, the values were 1.2±0.2, 3.6±0.9, 4.5±1.3 and 3.2±0.6, respectively. Significant differences were observed between the TLR2^-/-^ and TLR9^-/-^ mouse groups and their respective negative control groups, as well as between the WT, TLR2^-/-^, TLR9^-/-^ and TLR2/9^-/-^ infected mouse groups.

In the assay to determine expression of the TLR9 in the trigeminal ganglia (Figure 1) of the WT and TLR2^-/-^ infected mice, the arbitrary units were 98.9±19.0 and 9.9±2.1, respectively. Significant differences were observed between the WT and TLR2^-/-^ infected mouse groups and their respective negative control groups, as well as between the WT and TLR2^-/-^ infected mouse groups. In the brains (Figure 2) from the same groups, the values were 1.3±0.2 and 11.7±3.0, respectively. Significant differences were observed between the WT and TLR2^-/-^ infected mouse groups.

Commentary on the results and discussion in Additional file 1: Figure S1.

Histology was performed with H&E staining of the trigeminal ganglia sections of the C57BL/6, TLR2^-/-^, TLR9^-/-^ and TLR2/9^-/-^ animals to analyze and distinguish the level of infiltration and trigeminal ganglia lesions caused by HSV-1 after 5 days of infection (Figure S1). The infected C57BL/6 mice presented an architecture relatively altered by the presence of a diffuse inflammatory infiltrate, with neuronal body alterations such as cytoplasmic edge enhancement and perinuclear vacuolization. The cellularity was increased, suggesting glial cell proliferation and the infiltration of mononuclear cells. In the infected TLR2^-/-^ mice, the infiltrate was more pronounced and diffuse compared with the infected C57BL/6 mice, and there was interstitial edema (arrows). We noted the presence of polymorphonuclear cells (insert, arrow) next to areas of neuronal degeneration and necrosis (asterisk). In the TLR9^-/-^ animals, the infiltrate was noticeably more intense than in the previous groups, with higher inflammation, as evidenced by neuronal degeneration, cytoplasmic acidophilia and dissolution of the cytoplasmic boundaries. The infiltrate was rich in polymorphonuclear cells (arrow). The signs in the infected TLR2/9^-/-^ mice were similar to those in the infected TLR9^-/-^ mice, with intense internal changes in the neuronal bodies (asterisk), which were surrounded by polymorphonuclear cells (arrow).

Commentary on the results and discussion in Additional file 3: Figure S2.

There was no statistically significant difference in the production of iNOS, in the amount of macrophages nor in the production of iNOS by macrophages between infected C57BL/6 and infected TLR2/9-/- mice. However, the graphs show a clear trend toward higher levels of iNOS, more macrophages and more iNOS producer macrophages in infected C57BL/6 mice than in infected TLR2/9-/- mice. We therefore believe that this trend is biologically relevant.
